# Therapeutic Prospects of *Psidium guajava* Leaves: An Antibacterial Assessment Against Clinically Important Pathogens

**DOI:** 10.1155/ijm/8276652

**Published:** 2025-12-13

**Authors:** Bibi Rafeena Ally-Charles, Ede Tyrell, Rebecca Khatun, Richard Lall, Bibi Yassin, Martin King, Devi Rajnarine, Basil Dey, Narita Singh, Charlan Abrams, Andrew Hutson, Karishma Jeeboo

**Affiliations:** ^1^ College of Medical Sciences, University of Guyana, Georgetown, Guyana, uog.edu.gy; ^2^ Faculty of Natural Sciences, University of Guyana, Georgetown, Guyana, uog.edu.gy

**Keywords:** antibacterial activity, antibiotics, leaf extracts, multidrug resistance, phytochemicals, *Psidium guajava*, zone of inhibition

## Abstract

**Background:**

The rising incidence of multidrug resistance and drug toxicity has prompted the search for complementary and alternative treatments for bacterial infections.

**Objective:**

This study aimed to screen for the phytochemical present in *Psidium guajava* leaves, to determine the antibacterial potential of *P. guajava* leaves, and to compare the effectiveness of the *P. guajava* leaves against current antibiotics.

**Methods:**

Dried pulverised *P. guajava* leaves were macerated using different solvents and then concentrated using a rotary evaporator. The extracts were screened for phytochemicals, namely, saponins, alkaloids, tannins, flavonoids, phenols, steroids and terpenoids, according to standard testing procedures. Antibacterial discs were prepared by soaking 6‐mm sterile filter paper discs in different concentrations of the various extracts. Antibacterial susceptibility testing was done using the Kirby–Bauer disc diffusion method.

**Results:**

Phytochemical screening confirmed the presence of all tested phytochemicals in *Psidium guajava* leaf extracts. The ethyl acetate extract (EAE) demonstrated significant antimicrobial activity at 100 mg/mL, showing large zones of inhibition (ZOIs) against *Staphylococcus aureus* (22.0 ± 6.1 mm), *Escherichia coli* (16.3 ± 0.9 mm) and *Pseudomonas aeruginosa* (15.0 ± 0.0 mm). The ethanolic extract (EE) also showed strong activity, with significant ZOI against *Klebsiella pneumoniae* (22.0 ± 4.3 mm) and *P. aeruginosa* (14.0 ± 1.0 mm). ZOI for the 100 mg/mL extracts against *S. aureus* were significantly larger than those for ceftazidime (19 mm), while those against *P. aeruginosa* exceeded tetracycline (9 mm) (*p* = 0.001). The MIC results confirmed the strength of the EE, with the lowest values: 3.1 mg/mL against *K. pneumoniae* ATCC and 6.3 mg/mL against *S. aureus*, *E. coli* and *P. aeruginosa*, possibly due to the presence of saponins.

**Conclusions:**

*P. guajava* leaves contain many phytochemicals which in turn possess great antibacterial activity and therefore have great potential as a novel complementary and alternative treatment to antibiotics.

## 1. Introduction

The *Psidium guajava* plant bears a fruit called Guava. This plant is a part of the *Myrtaceae* family that grows in tropical and subtropical areas around the world [1]. The fruit is a rich source of vitamin C and is well known for its flavour, aroma and taste [2]. *P. guajava* has been traditionally used to treat a wide range of communicable and non‐communicable conditions. A decoction of the leaves and bark is used to treat diarrhoea, dysentery, diabetes, sore throat, menstrual cramps and bleeding gums [3]. *P. guajava* is also used to manage hypertension, especially in some parts of Africa [4].

*P. guajava* is thought to contain many bioactive compounds, also known as phytochemicals, with great medicinal benefits [5]. The phytochemical composition in *P. guajava* varies slightly depending on the geographical area in which they are grown and species to specific biochemical interactions [6]. *P. guajava* leaves contain several bioactive compounds such as saponins, tannins, alkaloids, flavonoids, anthraquinones and cardiac glycosides [7]. These phytochemicals are believed to be associated with various medicinal benefits of the *P. guajava* plant [8].

Medicinal plants have been given much attention recently because of the rise in the incidence and prevalence of infectious diseases [9] and the rise in multidrug‐resistant (MDR) pathogens to modern pharmacological treatments [10]. MDR strains are emerging, and this poses a threat to the pharmaceutical industry. Furthermore, many conventional antibiotics have severe side effects [11]. Currently, treatment for *Klebsiella* spp., *Escherichia coli*, *Staphylococcus* spp. and *Pseudomonas* spp., is becoming more difficult [12]. Therefore, scientists must explore other antimicrobial agents such as plant extracts to combat these bacteria [13].

Because of the increasing need for complementary and alternative treatment, *P. guajava* leaves have been extensively researched to explore their phytochemical composition and their effects in treating certain bacterial infections [14, 15]. *P. guajava* leaves have demonstrated antimicrobial activity against the bacteria *Bacillus* spp., *Clostridium* spp. and *Shigella* spp. [16, 17]; however, it is unclear whether the subspecies of *P. guajava* found on the coast of Guyana has antibacterial activity. Furthermore, a detailed analysis of its activity against other bacteria is necessary including against MDR strains of *Pseudomonas aeruginosa* and extended spectrum beta lactamases (ESBL) strains of *Klebsiella pneumoniae*.

There are many scientific articles available about the variety of plant species native to Guyana and their potential effects on infectious agents. However, a review of the literature showed that the antibacterial potential of *P. guajava* leaves was never done in Guyana. The investigators therefore sought to examine the antibacterial potential and phytochemicals present in *P. guajava* leaves and determine whether there were any differences between the antibacterial effects of the *P. guajava* leaf extracts and conventional antibiotics.

## 2. Methods

### 2.1. Preparation of Plant Extracts

**Figure 1:**
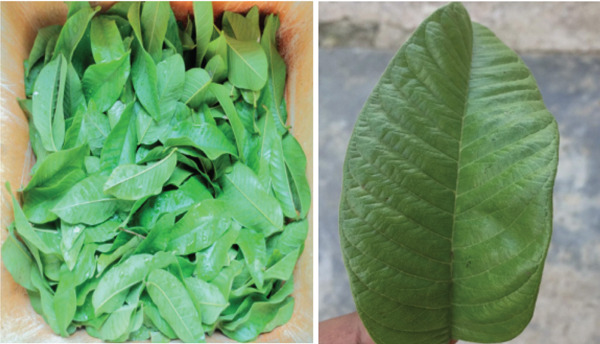
Freshly picked, healthy leaves from *Psidium guajava* (guava) plants.

Healthy *P. guajava* leaves (Figure 1) were gathered from the coastal regions in Guyana. The leaves were authenticated by the Biological Diversity Center at the University of Guyana. *P. guajava* leaves were thoroughly washed and dried at 28°C. The leaves were placed on a clean surface and allowed to dry for 1 month with occasional flipping. Dried *P. guajava* leaves were then pulverised in a sterilised mixer and transferred to sterile containers. The powdered leaves were then extracted at a weight‐to‐volume ratio of 1:5 (w/v), whereby 100 g of powdered plant material was macerated in 500 mL of each solvent: hexane (non‐polar), ethyl acetate (semi‐polar), methanol (polar) and 95% ethanol (polar). The solvents used in this study were selected based on their polarity indices to optimise the extraction of a wide range of phytochemicals. Maceration was carried out in dark bottles at room temperature for 72 h with intermittent shaking. The mixtures were then filtered using Whatman No. 1 filter paper every 24 h, and the filtrates were combined and then concentrated under reduced pressure using a rotary evaporator at 45°C [18].

### 2.2. Phytochemical Screening

The four crude extracts, that is, ethyl acetate extract (EAE), ethanolic extract (EE), methanolic extract (ME) and hexane extract (HE), were screened for common phytochemicals, namely, saponins, alkaloids, tannins, flavonoids, phenols, steroids and terpenoids, according to standard testing procedures [17].

#### 2.2.1. Saponins

Half a millilitre of EE was agitated with 2 mL of distilled water in a test tube. The formation of foams for 10 min indicated a positive test [17].

#### 2.2.2. Alkaloids

Two millilitres of EE was placed in a test tube, and a few drops of 2% picric acid solution was applied. The formation of an orange colour confirmed a positive test [17].

#### 2.2.3. Tannins

The EE was diluted with distilled water, and then a few drops 10% ferric chloride solution was added. The formation of a blue colour confirmed gallic tannins, while a green colour suggested catechol tannins [17].

#### 2.2.4. Flavonoids

Four millilitres of EE was mixed with 1.5 mL of methanolic solution (50%) in a test tube. The mixture was heated and metal magnesium was applied. Then, concentrated hydrochloric acid was added drop by drop to the mixture. Flavonoids’ presence was confirmed by the formation of a red colour [17].

#### 2.2.5. Phenols

One millilitre of EE was placed in a test tube, and a few drops of ferric chloride solution was applied. Phenols’ presence was confirmed by a bluish‐black colour formation [17].

#### 2.2.6. Steroids

One millilitre of EE was placed in a test tube with equal parts of acetic anhydride and chloroform to make up 2 mL. Concentrated sulphuric acid was then applied slowly. The presence of steroids was confirmed by a green bluish colour formation [17].

#### 2.2.7. Terpenoids

Some EE were placed in a test tube with 1 mL of acetic anhydride. Then, a few drops of concentrated sulphuric acid were added. Terpenoids’ presence was confirmed by a blue colour formation [17].

Qualitative analysis of these seven phytochemical was performed on the other three extracts (EAE, ME, HE) via the same methods described above.

### 2.3. Antibacterial Susceptibility Testing

#### 2.3.1. Preparation of Media

Mueller–Hilton agar (MHA) was prepared using dehydrated powder and distilled water. Thirty‐eight grams of dehydrated powder was reconstituted with 1 L of distilled water and heated with frequent shaken to fully dissolve all components. Sterilisation of MHA was achieved by autoclaving at 121°C for 15 min. The autoclaved media was brought down to room temperature and then poured into sterile petri plates. The liquefied media was allowed to solidify in a sterile area and then stored in a refrigerator until needed for antibacterial susceptibility testing.

#### 2.3.2. Test Organisms

The bacteria used were KWIK‐STIK *K. pneumoniae* American Type Culture Collection (ATCC) 700603, *Staphylococcus aureus* ATCC 25923, *Escherichia coli* ATCC 25922 and *P. aeruginosa* ATCC 27853. The bacteria were sourced from the United States (US) and were revived according to the manufacturer’s instructions. An ESBL *K. pneumoniae* (in‐house strain) was also used. This ESBL strain was collected from the Microbiology Department of the Georgetown Public Hospital Corporation (GPHC). These bacteria were chosen because they are clinically important pathogens, frequently associated with hospital‐ and community‐acquired infections. They represent Gram‐positive and Gram‐negative bacteria, including multidrug‐resistant strains, making them suitable models to test the antibacterial potential of *Psidium guajava* leaf extracts in the context of current therapeutic challenges.

#### 2.3.3. Preparation of *P. guajava* Treatments

Concentrations of 5 mL each of 100 mg/mL (crude extract), 50 mg/mL, 25 mg/mL and 12.5 mg/mL were prepared via serial dilution using the stock solutions of 95% ethanol, methanol, ethyl acetate and hexane with their respective crude extracts. Six‐millimetre antibacterial discs were prepared using sterile Whatman No. 3 filter papers soaked in varying concentrations of the different leaf extracts overnight. These treatment discs were then used to perform antibacterial susceptibility testing with the known bacteria.

#### 2.3.4. Preparation of Standard (Controls) Treatments

To compare the performance of the leaf extracts, the antibiotic ciprofloxacin (CIP), ceftazidime (CAZ) and tetracycline (TE), 5 *μ*g each, was used as the positive controls. These discs were purchased in a ready‐to‐use form. The negative controls were prepared by submerging sterile 6‐mm Whatman No. 3 filter paper discs in the various solvents overnight.

#### 2.3.5. Sensitivity Testing

The Kirby–Bauer disc diffusion technique was carried out on MHA following Clinical Laboratory Standards Institute (CLSI) guidelines. The treatment discs from a particular extract concentration were placed in triplicates on the MHA plate after being seeded with an appropriate test bacteria. Incubated was carried out at 37°C for 24 h. Zone of inhibitions (ZOIs) were measured in mm and validated by two microbiologists. The ZOI was recorded in Statistical Package for the Social Sciences (SPSS) for data analysis. For this study, a mean ZOI ≥ 10.0 mm was considered an effective treatment.

The minimum inhibitory concentration (MIC) is the lowest concentration of an antibacterial agent that prevents visible growth of a bacteria. The MIC in this study was estimated via a method adopted by Faujdar et al. [19].

### 2.4. Data Analysis

All data analyses, including means and standard deviation (SD), were calculated using SPSS version 20. The one‐way analysis of variance (ANOVA) tests were done to determine the statistical differences among the ZOIs for the various treatments (*p* < 0.05). The least significance difference (LSD) test was used to perform multiple comparisons among the various treatments.

## 3. Results

### 3.1. Phytochemicals Present in *P. guajava* Leaf Extracts

**Figure 2:**
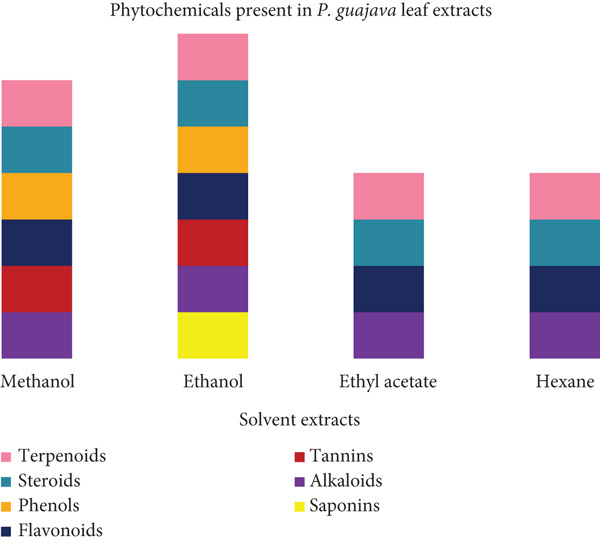
Presence of phytochemical compounds in different *Psidium guajava* leaf extracts.

Figure 2 shows that all seven of the phytochemicals that we tested for were present in the EE, while six were present in the ME with saponins being absent. Four of the phytochemicals that we screened for were present in the EAE and HE, with tannins, saponins and phenols being absent.

### 3.2. Activity of *P. guajava* EAE Against the Test Organisms

The EAE exhibited a dose‐dependent antibacterial activity across all the test bacteria. At the highest concentration (100 mg/mL), EAE showed the largest ZOI against *S. aureus* (22.00 ± 6.08 mm), followed by *E. coli* (16.33 ± 1.15 mm) (Figure 3a), *P. aeruginosa* (15.00 ± 0.00 mm), and *K. pneumoniae* ESBL (10.33 ± 0.58 mm). The lowest activity at this concentration was observed against *K. pneumoniae* ATCC (9.00 ± 0.00 mm). Activity progressively decreased with lower concentrations, and little to no inhibition was observed at 12.5 mg/mL across all bacterial species (Table 1).

Figure 3Zones of inhibition observed in antibacterial susceptibility testing of selected bacteria.

**Figure:**
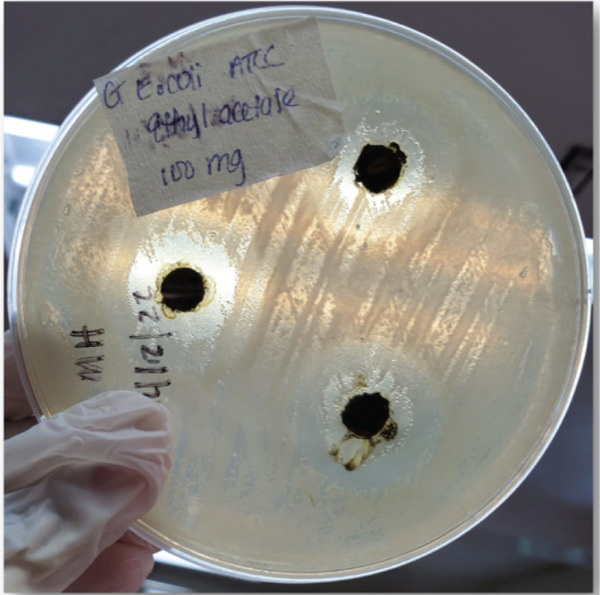
(a) *E. coli* ATCC at 100 mg/mL (EAE)

**Figure:**
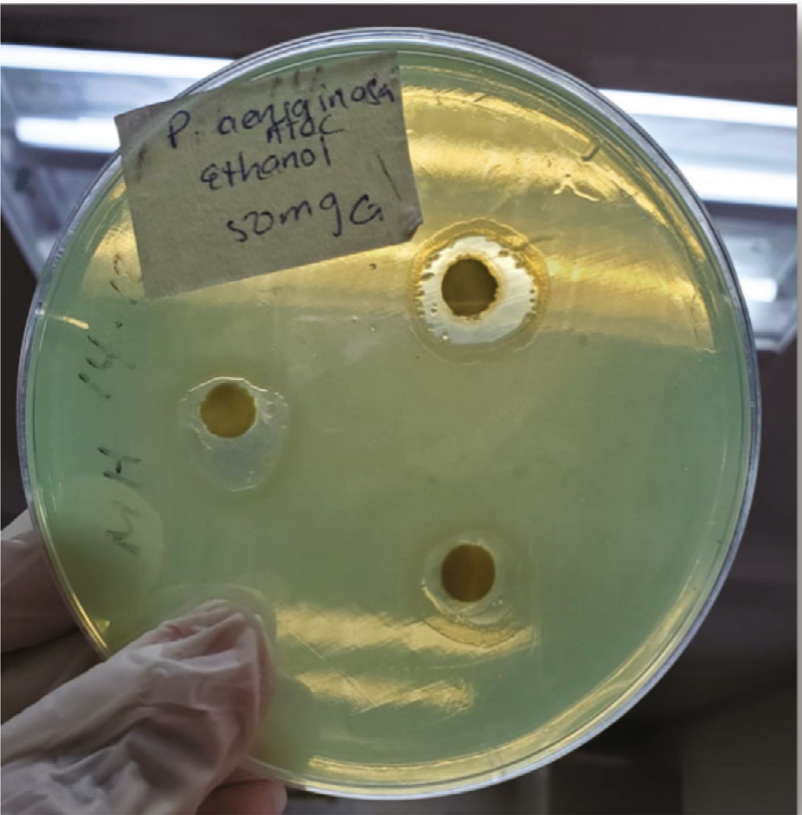
(b) *P. aeruginosa* at 50 mg/mL (EE)

**Figure:**
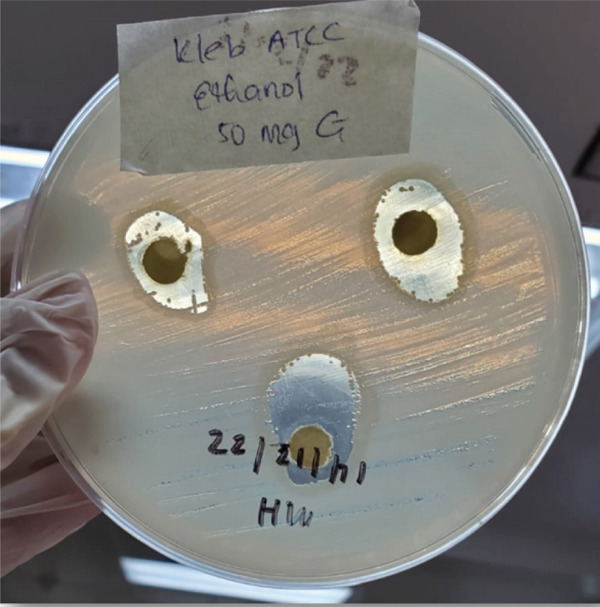
(c) *K. pneumoniae* ATCC at 50 mg/mL (EE)

**Figure:**
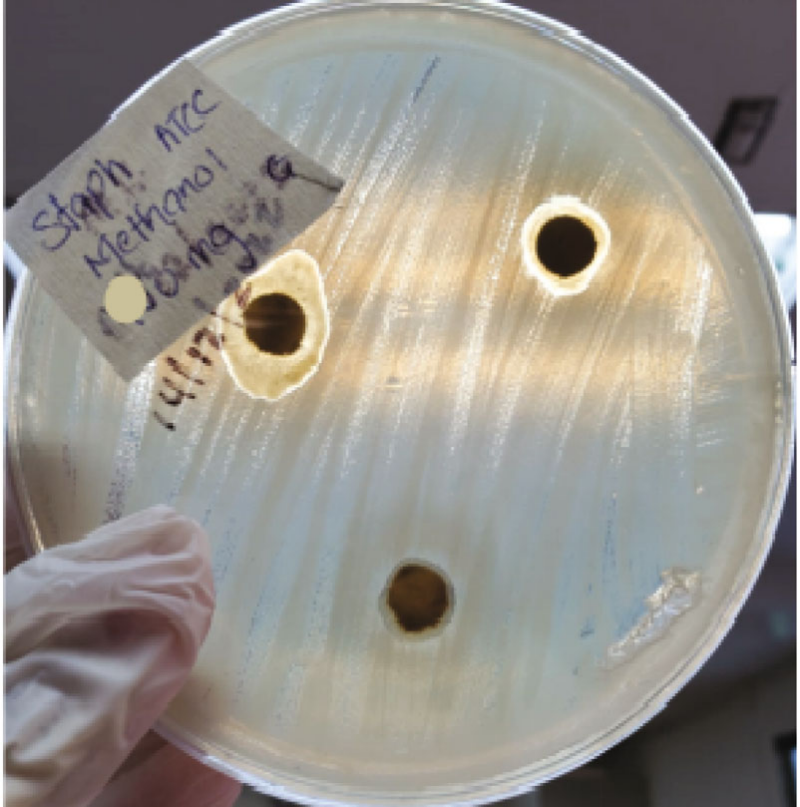
(d) *S. aureus* at 50 mg/mL (ME)

**Table 1:** The activity of *P. guajava* EAE against the test organisms.

**Treatments**	**Average ZOI for the bacteria (mm)**				
	***K. pneumoniae* ATCC**	***K. pneumoniae* ESBL**	***S. aureus***	***E. coli***	***P. aeruginosa***
100 mg/mL	9.00 ± 0.00^d^	10.33 ± 0.58^c^	22.00 ± 6.08b^c^	16.33 ± 1.15^d^	15.00 ± 0.00^c^
50 mg/mL	8.00 ± 0.00^d^	6.00 ± 0.00^d^	9.00 ± 0.00^d^	10.67 ± 0.58^e^	11.00 ± 0.00^d^
25 mg/mL	6.50 ± 0.00^e^	6.00 ± 0.00^d^	8.00 ± 2.00^d^	10.33 ± 1.53^e^	7.00 ± 0.00^f^
12.5 mg/mL	6.00 ± 0.00^e^	6.00 ± 0.00^d^	7.00 ± 1.73^d^	7.67 ± 1.15^f^	6.00 ± 0.00^f^
CIP	27.00 ± 0.00^a^	15.00 ± 0.00^b^	27.00 ± 0.00^ab^	39.00 ± 0.00^a^	30.67 ± 1.15^a^
CAZ	13.00 ± 0.00^c^	19.00 ± 0.00^a^	19.00 ± 0.00^c^	30.00 ± 0.00^b^	29.33 ± 0.58^b^
TE	17.00 ± 0.00^b^	6.00 ± 0.00^d^	28.67 ± 1.15^a^	22.00 ± 0.00^c^	9.33 ± 1.15^e^
*p* value	0.001	0.001	0.001	0.001	0.001

*Note:* Data with the same superscript letters are statistically similar.

Among the standard antibiotics, CIP showed the highest overall activity, with ZOI values ranging from 15.00 ± 0.00 mm (*K. pneumoniae* ESBL) to 39.00 ± 0.00 mm (*E. coli*). TE was most effective against *S. aureus* (28.67 ± 1.15 mm) but showed no activity against *K. pneumoniae* ESBL (6.00 ± 0.00 mm). CAZ showed strong activity against *K. pneumoniae* ESBL (19.00 ± 0.00 mm) and *P. aeruginosa* (29.33 ± 0.58 mm), outperforming EAE at all concentrations (Table 1).

Statistical analysis indicated significant differences in antibacterial activity among the treatments for all tested bacteria (*p* = 0.001 for each strain). Superscript letters in the table denote significant differences in ZOI values within each bacterial column (LSD, *p* < 0.05) (Table 1).

Our findings suggest that the EAE possesses moderate antibacterial properties, particularly against *S. aureus* and *E. coli*, with potential for further development. However, its efficacy is lower than that of conventional antibiotics.

### 3.3. Activity of *P. guajava* EE Against the Test Organisms

The EE demonstrated dose‐dependent activity against *K. pneumoniae* ATCC, *S. aureus*, *E. coli* and *P. aeruginosa*. At the highest concentration (100 mg/mL), the extract produced the largest ZOI against *K. pneumoniae* ATCC (22.00 ± 5.29 mm), followed by *P. aeruginosa* (14.00 ± 1.00 mm), *S. aureus* (10.50 ± 0.50 mm) and *E. coli* (10.67 ± 0.58 mm). No dose‐dependent response was observed for *K. pneumoniae* ESBL, as the ZOI remained constant (6.00 ± 0.00 mm) across all EAE concentrations (Table 2). ZOIs for *P. aeruginosa* and *K. pneumoniae* ATCC at 50 mg/mL concentrations are shown in Figure 3b,c, respectively.

**Table 2:** The activity of *P. guajava* EE against the test organisms.

	**Average ZOI for the bacteria (mm)**				
	***K. pneumoniae* ATCC**	***K. pneumoniae* ESBL**	***S. aureus***	***E. coli***	***P. aeruginosa***
100 mg/mL	22.00 ± 5.29^ab^	6.00 ± 0.00^c^	10.50 ± 0.50^c^	10.67 ± 1.53^d^	14.00 ± 1.00^b^
50 mg/mL	15.00 ± 5.00^c^	6.00 ± 0.00^c^	9.67 ± 0.58^c^	9.33 ± 1.15^de^	10.33 ± 0.58^c^
25 mg/mL	13.33 ± 1.53^cd^	6.00 ± 0.00^c^	9.33 ± 1.15^c^	9.33 ± 1.53^de^	9.33 ± 0.58^cd^
12.5 mg/mL	10.33 ± 2.52^d^	6.00 ± 0.00^c^	9.33 ± 0.58^c^	8.67 ± 0.58^e^	8.67 ± 0.58^d^
CIP	27.00 ± 0.00^a^	15.00 ± 0.00^b^	27.00 ± 0.00^a^	39.00 ± 0.00^a^	30.67 ± 1.15^a^
CAZ	13.00 ± 0.00^cd^	19.00 ± 0.00^a^	19.00 ± 0.00^b^	30.00 ± 0.00^b^	29.33 ± 0.58^a^
TE	17.00 ± 0.00b^c^	6.00 ± 0.00^c^	28.67 ± 1.15^a^	22.00 ± 0.00^c^	9.33 ± 1.15^cd^
*p* value	0.001	0.001	0.001	0.001	0.001

*Note:* Data with the same superscript letters are statistically similar.

Among the standard antibiotics, CIP exhibited the highest overall activity, with ZOIs ranging from 15.00 ± 0.00 mm (*K. pneumoniae* ESBL) to 39.00 ± 0.00 mm (*E. coli*). TE showed strong activity against *S. aureus* (28.67 ± 1.15 mm) and *E. coli* (22.00 ± 0.00 mm) but no activity against *K. pneumoniae* ESBL (6.00 ± 0.00 mm) and low activity against *P. aeruginosa* (9.33 ± 1.15 mm). CAZ was most effective against *K. pneumoniae* ESBL (19.00 ± 0.00 mm) and *P. aeruginosa* (29.33 ± 0.58 mm), though it had reduced activity against *K. pneumoniae* ATCC (13.00 ± 0.00 mm) (Table 2).

Statistical analysis revealed significant differences in antibacterial activity among treatments for all tested bacterial strains (*p* = 0.001). LSD analysis indicated that the ZOI values marked with different superscript letters differ significantly (*p* < 0.05) (Table 2).

Overall, the EAE showed promising antibacterial effects, particularly against *K. pneumoniae* ATCC and *P. aeruginosa*, although its activity was lower than that of the standard antibiotics.

### 3.4. Activity of *P. guajava* ME Against the Test Organisms

The extract showed moderate antibacterial activity with a concentration‐dependent trend against *K. pneumoniae* ATCC, *S. aureus*, *E. coli* and *P. aeruginosa*. The highest ZOI was observed at 100 mg/mL for *S. aureus* (17.00 ± 0.00 mm), followed by *K. pneumoniae* ATCC (11.67 ± 0.58 mm) and *E. coli* (11.33 ± 2.31 mm). Activity against *P. aeruginosa* was comparatively lower, with a ZOI of 8.33 ± 0.58 mm at the highest concentration. Conversely, *K. pneumoniae* ESBL remained resistant across all concentrations of the extract, showing no inhibition at any tested dose (Table 3). ZOIs for *S. aureus* at 50 mg/mL concentration is shown in Figure 3d.

**Table 3:** The activity of *P. guajava* ME against the test organisms.

	**Average ZOI for the bacteria (mm)**				
	***K. pneumoniae* ATCC**	***K. pneumoniae* ESBL**	***S. aureus***	***E. coli***	***P. aeruginosa***
100 mg/mL	11.67 ± 0.58^c^	6.00 ± 0.00^c^	17.00 ± 0.00^c^	11.33 ± 2.31^d^	8.33 ± 0.58^b^
50 mg/mL	9.33 ± 2.31^d^	6.00 ± 0.00^c^	12.33 ± 2.08^e^	9.33 ± 1.15^de^	7.00 ± 0.00^d^
25 mg/mL	9.00 ± 1.00^d^	6.00 ± 0.00^c^	9.00 ± 0.00^f^	8.33 ± 1.53^e^	6.00 ± 0.00^d^
12.5 mg/mL	6.00 ± 0.00^e^	6.00 ± 0.00^c^	8.33 ± 0.58^f^	7.33 ± 0.58^e^	6.00 ± 0.00^d^
CIP	27.00 ± 0.00^a^	15.00 ± 0.00^b^	27.00 ± 0.00^a^	39.00 ± 0.00^a^	30.67 ± 1.15^a^
CAZ	13.00 ± 0.00^c^	19.00 ± 0.00^a^	19.00 ± 0.00^b^	30.00 ± 0.00^b^	29.33 ± 0.58^a^
TE	17.00 ± 0.00^b^	6.00 ± 0.00^c^	28.67 ± 1.15^a^	22.00 ± 0.00^c^	9.33 ± 1.15^b^
*p* value	0.001	0.001	0.001	0.001	0.001

*Note:* Data with the same superscript letters are statistically similar.

Among the standard antibiotics, CIP displayed the highest antibacterial efficacy, producing ZOI ranging from 15.00 ± 0.00 mm (*K. pneumoniae* ESBL) to 39.00 ± 0.00 mm (*E. coli*). Similarly, CAZ showed notable activity against *K. pneumoniae* ESBL (19.00 ± 0.00 mm) and *P. aeruginosa* (29.33 ± 0.58 mm), while TE was most effective against *S. aureus* (28.67 ± 1.15 mm) and *E. coli* (22.00 ± 0.00 mm) (Table 3).

Statistical analysis revealed significant differences among treatments for all bacterial strains tested (*p* = 0.001). Superscript letters in Table 3 indicate statistically significant differences within each bacterial group (*p* < 0.05).

Our results demonstrate that *P. guajava* ME exhibits antibacterial potential particularly against susceptible strains, though it is less effective against MDR *K. pneumoniae* ESBL.

### 3.5. Activity of *P. guajava* HE Against the Test Organisms

The HE showed minimal antibacterial activity at all concentrations tested, with ZOI remaining constant at 6.00 ± 0.00 mm for *K. pneumoniae* ATCC, *K. pneumoniae* ESBL and *P. aeruginosa* across all concentrations. Slightly higher activity was observed against *S. aureus* and *E. coli* at 100 mg/mL, with ZOIs of 8.67 ± 0.58 mm for both strains (Table 4).

**Table 4:** The activity of *P. guajava* HE against the test organisms.

	**Average ZOI for the bacteria (mm)**				
	***K. pneumoniae* ATCC**	***K. pneumoniae* ESBL**	***S. aureus***	***E. coli***	***P. aeruginosa***
100 mg/mL	6.00 ± 0.00^d^	6.00 ± 0.00^c^	8.67 ± 0.58^d^	8.67 ± 0.58^d^	6.00 ± 0.00^d^
50 mg/mL	6.00 ± 0.00^d^	6.00 ± 0.00^c^	6.00 ± 0.00^e^	6.00 ± 0.00^e^	6.00 ± 0.00^d^
25 mg/mL	6.00 ± 0.00^d^	6.00 ± 0.00^c^	6.00 ± 0.00^e^	6.00 ± 0.00^e^	6.00 ± 0.00^d^
2.5 mg/mL	6.00 ± 0.00^d^	6.00 ± 0.00^c^	6.00 ± 0.00^e^	6.00 ± 0.00^e^	6.00 ± 0.00^d^
CIP	27.00 ± 0.00^a^	15.00 ± 0.00^b^	27.00 ± 0.00^b^	39.00 ± 0.00^a^	30.67 ± 1.15^a^
CAZ	13.00 ± 0.00^c^	19.00 ± 0.00^a^	19.00 ± 0.00^c^	30.00 ± 0.00^b^	29.33 ± 0.58^b^
TE	17.00 ± 0.00^b^	6.00 ± 0.00^c^	28.67 ± 1.15^a^	22.00 ± 0.00^c^	9.33 ± 1.15^c^
*p* value	0.001	0.001	0.001	0.001	0.001

*Note:* Data with the same superscript letters are statistically similar.

Standard antibiotics demonstrated significantly higher antibacterial efficacy compared to the HE. CIP exhibited the greatest activity, producing ZOIs ranging from 15.00 ± 0.00 mm (*K. pneumoniae* ESBL) to 39.00 ± 0.00 mm (*E. coli*). CAZ showed notable activity, particularly against *K. pneumoniae* ESBL (19.00 ± 0.00 mm), *S. aureus* (19.00 ± 0.00 mm) and *E. coli* (30.00 ± 0.00 mm). TE was most effective against *S. aureus* (28.67 ± 1.15 mm) and *E. coli* (22.00 ± 0.00 mm) (Table 4).

Statistical analysis confirmed significant differences among treatments for all bacteria tested (*p* = 0.001). The low antibacterial activity of the HE indicates limited effectiveness against the tested bacterial strains compared to standard antibiotics.

Notably, no zones of inhibition were observed for the negative control discs; therefore, these results have been omitted from all the tables.

Table 5 reveals the MIC values of the *P. guajava* leaf treatments against the test organism. The EE demonstrated the highest antibacterial activity, with the lowest MIC values recorded against *K. pneumoniae* ATCC (3.1 mg/mL), *S. aureus*, *E. coli* and *P. aeruginosa* (6.3 mg/mL each). The EAE also showed appreciable activity, with MIC values of 6.3 mg/mL against *E. coli* and *S. aureus*, and 12.5 mg/mL against *K. pneumoniae* ATCC and *P. aeruginosa.* The ME showed similar activity against *K. pneumoniae* ATCC, *S. aureus* and *E. coli* (12.5–6.3 mg/mL), but was less effective against *P. aeruginosa* (25 mg/mL). The HE displayed the weakest antibacterial activity, with consistently high MIC values (50–100 mg/mL) across all tested organisms. All extracts were markedly less effective against the ESBL‐producing *K. pneumoniae*, with MICs ranging from 50 mg/mL (EAE) to 100 mg/mL (EE, ME, HE).

**Table 5:** MIC of *P. guajava* leaf treatments against the test organisms.

**Treatments**	**MIC (mg/mL)**				
	***K. pneumoniae* ATCC**	***K. pneumoniae* ESBL**	***S. aureus***	***E. coli***	***P. aeruginosa***
EAE	12.5	50	6.3	6.3	12.5
EE	3.1	100	6.3	6.3	6.3
ME	12.5	100	6.3	6.3	25
HE	100	100	50	50	100

## 4. Discussion

The medicinal importance of phytochemicals in plants such as *Psidium guajava* cannot be overstated, given their wide range of bioactive properties. In this study, seven major classes of phytochemicals were screened across four different *P. guajava* leaf extracts. The EE contained all seven phytochemicals, highlighting its richness in secondary metabolites. This finding contrasts with previous reports. For example, Ekeleme et al. (2017) reported the presence of flavonoids, alkaloids, tannins, and terpenoids in ethanol extracts of *P. guajava* leaves collected in Nigeria but noted the absence of saponins. They did not test for phenols and steroids, although they reported a high concentration of flavonoids [17]. Similarly, Dhilip et al. (2015) identified all the phytochemicals we screened for, except saponins, in *P. guajava* leaves from India; however, terpenoids were not evaluated in their study [20]. In another study conducted in the United States, Biswas et al. (2013) reported the presence of flavonoids, tannins, terpenoids and phenols, while saponins were absent [15].

Interestingly, the absence of saponins was a consistent finding across all three studies but contrasts with our results, where saponins were detected. This inconsistency may reflect geographical variations, differences in extraction methods or seasonal factors influencing phytochemical composition.

Ngene et al. (2019) reported the presence of all seven phytochemicals assessed in this study, along with additional compounds, in *P. guajava* leaves from Nigeria. Notably, high concentrations of tannins, alkaloids and saponins were detected [21]. Another Nigerian study also identified six of the seven phytochemicals screened in our research, along with other metabolites; however, steroids were absent [22]. This contrasts with our findings, where steroids were present. The consistent presence of saponins in both Nigerian studies aligns with our results and reinforces the likelihood of their occurrence in *P. guajava* leaves from this region.

In contrast, a study conducted in India reported that the EE contained phenols, flavonoids and tannins, but lacked terpenoids [23], which is inconsistent with our findings. Their study also noted that tannins were present at the highest concentration. While we did not quantify phytochemicals in our study, we postulate that the EE may contain high levels of flavonoids and tannins based on qualitative observations.

In the current study, the ME contained all tested phytochemicals except saponins. A related study from Uganda found saponins and four other phytochemicals in the ME, which partially overlaps with our findings; however, alkaloids and steroids were not evaluated in that study [4]. A Nigerian study similarly reported the presence of saponins, tannins, flavonoids and terpenoids in the ME, but noted the absence of alkaloids [17], which contradicts our findings where alkaloids were present. In Libya, another investigation reported the presence of five of the phytochemicals we screened for in the ME, although steroids and phenols were not assessed [24]. The consistent detection of saponins in the ME across these studies contrasts with our results, where saponins were not observed. Additionally, an Indian study found phenols, flavonoids, tannins, and terpenoids in the ME, with tannins reported at the highest concentration [23]. Although we did not perform quantitative analysis, we hypothesise that the ME in our study also contains elevated levels of certain component, particularly tannins.

Our study revealed that the EAE and HE of *P. guajava* leaves lacked tannins, saponins and phenols. This finding contrasts with a study conducted in India, which reported the presence of tannins in the EAE [25]. Another Indian study identified six of the phytochemicals screened in our study within the EAE, along with additional compounds; however, flavonoids were notably absent in their results [26]. Interestingly, the phytochemicals that were absent in our EAE extracts, tannins, saponins and phenols, were all detected in their study. A further investigation in India found all seven phytochemicals screened in our study present in the EAE, although phenols were not tested in their study [27].

Comparative data on HE of *P. guajava* leaves are limited. However, a study conducted in the United States reported that none of the screened phytochemicals, namely, flavonoids, tannins, terpenoids, phenols or saponins, were present in the HE [15]. This contrasts with our findings, in which flavonoids and terpenoids were detected.

Phytochemical variation in medicinal plants is influenced by a range of spatial and temporal environmental factors. Abiotic stress is one such factor that has been shown to reduce phytochemical yield. Stressors such as drought, flooding, temperature extremes, variations in light intensity, soil quality and exposure to toxic substances can induce secondary metabolic responses in plants, leading to fluctuations in phytochemical composition [28]. In the present study, the *P. guajava* leaves were harvested from plants grown in uncontrolled environmental conditions. This limitation is also common in many previously published phytochemical studies. Thus, environmental variability may have played a significant role in the differences observed across studies, although many reports consistently identify a core group of phytochemicals in *P. guajava* leaves.

The present study found that the semi‐polar EAE exhibited antibacterial activity with ZOI greater than 10 mm for four of the five test organisms at 100 mg/mL. The extract was ineffective against *K. pneumoniae* ESBL but showed the highest activity against *S. aureus* at the same concentration. In a similar study conducted in Uttarakhand, India, comparable ZOIs were reported for *S. aureus* (21 mm) and *E. coli* (15 mm) using the EAE at 100 mg/mL [27]. However, their reported ZOI for *P. aeruginosa* (22 mm) was substantially higher than that observed in the present study. Another Indian study from Uttar Pradesh also reported a similar ZOI for *S. aureus* (21.5 mm) but larger zones for *E. coli* (22 mm) and *P. aeruginosa* (22.5 mm) [26]. The ZOIs for *E. coli* and *P. aeruginosa* were consistent across these two Indian studies but differed from our findings. This inconsistency may be attributed to geographical variation, which can influence the phytochemical composition of the plant extracts and, consequently, their antibacterial activity.

In our study, the EE demonstrated antibacterial activity with ZOIs exceeding 10 mm for four bacterial strains at 100 mg/mL. A study from Nigeria similarly reported that the EE was effective against four of the same test organisms [17]. However, while our results showed that the EE was ineffective against *K. pneumoniae* ESBL, it showed the greatest activity against *K. pneumoniae* ATCC at 100 mg/mL. In contrast, the Nigerian study found the EE to be most effective against *E. coli*, with a ZOI of 16 mm at the highest concentration tested (50 mg/mL), which was notably higher than the ZOI observed in our study at the same concentration [17]. Another Nigerian study reported a similar ZOI for *S. aureus* (10 mm), a smaller ZOI for *K. pneumoniae* (11.5 mm), and a larger ZOI for *P. aeruginosa* (12.5 mm) compared to our 50 mg/mL findings [17]. Additional studies reported ZOIs of 11 mm [15] and 18.3 mm [22] for *S. aureus* at 100 mg/mL of EE. Furthermore, another Nigerian study found larger ZOIs for *S. aureus* (16 mm) and *E. coli* (22 mm) when using 80 mg/mL of the EE [21].

Multiple studies have shown that the EE is effective against *K. pneumoniae* [17, 29], an effect often attributed to the presence of flavonoids [30]. Based on our findings, we hypothesise that the EE contains higher levels of flavonoids than the other extracts tested. Supporting this, Srivastava and Singh (2022) reported considerably higher ZOIs using the EE for *P. aeruginosa* (16.5 mm), *S. aureus* (20 mm) and *E. coli* (18.5 mm) compared to the results observed in our study [31].

In this study, the ME demonstrated antibacterial activity with ZOI greater than 10 mm against three of the five test organisms at 100 mg/mL. The extract was ineffective against *K. pneumoniae* ESBL and *P. aeruginosa*, but showed the highest activity against *S. aureus*. The ZOI for *S. aureus* in our study was consistent with the findings from a study conducted in Libya, where zones ranging from 13 to 17 mm were reported [24], but slightly higher than the value recorded in a US study (12.3 mm) [15]. A Nigerian study found that the ME was most effective against *K. pneumoniae*, with a ZOI of 16.5 mm at 50 mg/mL, substantially greater than what we observed [17]. That same study reported slightly higher ZOIs for *S. aureus* (14 mm) and *P. aeruginosa* (14 mm), while *E. coli* was resistant. In contrast, our ME showed a small but measurable zone against *E. coli*.

Srivastava and Singh (2022) also reported significantly larger ZOIs for *P. aeruginosa* (22 mm), *S. aureus* (23.5 mm) and *E. coli* (22.5 mm) when compared to our ME results [31]. For the HE, a prior study reported no inhibition against *S. aureus* and *E. coli* [15], whereas our study detected a very small ZOI for these organisms. Overall, our findings showed that the EE was more effective than the ME, which contrasts with the results reported by Srivastava and Singh (2022), where the ME demonstrated superior activity [31].

Our study also highlighted important differences in effectiveness between plant extracts and conventional antibiotics. Notably, resistance to tetracycline was observed in both *K. pneumoniae* ESBL and *P. aeruginosa*. In contrast, both the EE and EAE showed effectiveness against *P. aeruginosa*, while the EE alone was active against *K. pneumoniae* ESBL. At 100 mg/mL, the ZOIs produced by both extracts were significantly larger than those observed for TE, an important and remarkable finding.

Interestingly, the EAE demonstrated greater antibacterial activity than ceftazidime, a third‐generation broad‐spectrum cephalosporin [32]. Given that *P. guajava* leaf extracts were effective against MDR strains such as ESBL‐producing *K. pneumoniae* and *P. aeruginosa*, this suggests potential for these natural extracts as alternative antibacterial agents, especially where conventional treatments fail [29].

Additionally, the EE was effective against *K. pneumoniae* ATCC at concentrations as low as 12.5 mg/mL and against *P. aeruginosa* up to 50 mg/mL. Similarly, the EAE was active against *E. coli* at 25 mg/mL and *P. aeruginosa* at 50 mg/mL. These results support a dose‐dependent relationship, where higher concentrations of extracts led to larger ZOIs, a trend consistent with several other studies [12, 17, 18].

Our results are encouraging, as the *P. guajava* leaf extracts demonstrated activity against both Gram‐positive bacteria (GPB) and Gram‐negative bacteria (GNB), suggesting that the bioactive metabolites present in the plant may possess broad‐spectrum antibacterial properties. This observation is supported by a study conducted in Nigeria, which also reported broad‐spectrum activity of *P. guajava* extracts [17]. However, our findings contrast with another study in which the ME and EE extracts of *P. guajava* leaves were only effective against GPB, while all solvent extracts were ineffective against GNB [16].

Interestingly, the EE was the only extract that contained saponins. We propose that the presence of saponins may have contributed to the observed antibacterial activity against the GNB strain *K. pneumoniae* ATCC. This hypothesis differs from previous findings, where saponins were reported to have inhibitory effects primarily on GPB, such as *S. aureus* [15]. Although both the EAE and HE shared the same profile of identified phytochemicals, the EAE displayed significantly greater antibacterial activity. This suggests that the EAE may either contain additional bioactive compounds not detected in our screening or possess a higher concentration of the detected metabolites.

While the ME did exhibit some antibacterial activity, its efficacy was generally lower than that of the EE and EAE, despite containing most of the phytochemicals screened. We hypothesise that this reduced activity may be due to lower concentrations of these bioactive constituents in the ME, which indicates the importance of phytochemical quantity, and not just presence, in determining antimicrobial potency.

The MIC results indicate that *P. guajava* leaf extracts exhibited variable antibacterial activity against the test organisms. EE exhibited the strongest activity, with MICs of 3.1 mg/mL against *K. pneumoniae* ATCC and 6.3 mg/mL against *S. aureus*, *E. coli* and *P. aeruginosa*. This may be due to saponins present only in EE, which likely contributed to its activity, conflicting with reports where saponins inhibited GPB [30].

EAE showed moderate activity, particularly against *S. aureus* and *E. coli* (6.3 mg/mL), despite having a similar metabolite profile to HE, suggesting either higher metabolite concentrations or the presence of untested bioactive compounds. ME demonstrated moderate inhibition (6.3–12.5 mg/mL) but was less effective against *P. aeruginosa* (25 mg/mL), possibly due to lower concentrations of its phytochemicals. All extracts were less effective against ESBL‐producing *K. pneumoniae* (50–100 mg/mL), highlighting the challenge of MDR strains and the need for further fractionation and quantification of active compounds.

While previous studies have established the antimicrobial potential of *Psidium guajava* leaf extracts, many have focused primarily on qualitative assessments or limited extract types and microbial strains. This study extends the current body of knowledge by evaluating and comparing the antibacterial activity of multiple solvent extracts, hexane, ethyl acetate, methanol and 95% ethanol, against both standard and MDR clinical pathogens, including ESBL‐producing *Klebsiella pneumoniae*. The inclusion of MIC estimation alongside disc diffusion assays provides a more nuanced understanding of the dose‐dependent effects of the extracts. Importantly, the study demonstrates that EE and ME were more effective than HE and EAE, reaffirming the critical role of solvent polarity in extracting active phytochemicals. Moreover, the absence of antibacterial activity in HE highlights the specificity of polar compounds in contributing to bioactivity. By integrating antimicrobial screening with statistical validation and preliminary MIC estimation, this study provides a more rigorous and comparative framework than many earlier reports. These findings lay the groundwork for further bioassay‐guided fractionation, metabolite quantification and in vivo studies to support the development of guava leaf‐based alternative therapies for bacterial infections, including those caused by resistant strains.

## 5. Conclusion

This study confirms that *P. guajava* leaf extracts possess great antibacterial activity, likely due to the presence of multiple phytochemicals with known antimicrobial properties. In some cases, the efficacy of the extracts was comparable to or exceeded that of selected conventional treatments, highlighting their potential as natural alternatives for the management of bacterial infections. These findings provide a scientific basis for the development of complementary and alternative therapeutic agents derived from guava leaves.

We recommend that future studies incorporate comprehensive phytochemical analyses, including the quantification of key metabolites, to identify the specific bioactive constituents responsible for the observed effects. In addition, the use of more precise antimicrobial testing methods, such as broth microdilution for accurate determination of MIC and MBC values, is strongly encouraged. In addition, time‐kill assays could further elucidate whether the extracts exert bactericidal or bacteriostatic effects. Moreover, bioassay‐guided fractionation, in vivo efficacy and toxicity studies using relevant animal models, and synergistic evaluations with conventional antibiotics are essential to fully validate the therapeutic potential and clinical applicability of these plant‐based extracts.

**Nomenclature**
ZOI: Zone of inhibition
MDR: Multidrug resistance
ATCC: American Type Culture Collection
CLSI: Clinical Laboratory Standards Institute
ESBL: Extended spectrum beta lactamases
EAE: Ethyl acetate extract
EE: Ethanolic extract
ME: Methanolic extract
HE: Hexane extract
CIP: Ciprofloxacin
CAZ: Ceftazidime
TE: Tetracycline
MHA: Mueller–Hilton agar
GPHC: Georgetown Public Hospital Corporation
SPSS: Statistical Package for the Social Sciences
GPB: Gram‐positive bacteria
GNB: Gram‐negative bacteria

## Ethics Statement

All laboratory procedures involving bacterial isolates, including MDR strains such as ESBL‐producing *K. pneumoniae* and *P. aeruginosa*, were performed in accordance with standard biosafety protocols under Biosafety Level 2 (BSL‐2) conditions. Aseptic techniques were strictly followed throughout all microbiological procedures to prevent cross‐contamination. Certified Class II biosafety cabinets were used for culture handling and antibacterial testing, and appropriate personal protective equipment (PPE) was worn at all times to ensure the safety of laboratory personnel and prevent environmental contamination. The proposal was submitted to the Ministry of Health Institutional Review Board (IRB), and approval was granted. No other ethical considerations were required.

## Conflicts of Interest

The authors declare no conflicts of interest.

## Author Contributions

Bibi Rafeena Ally‐Charles and Ede Tyrell conceptualised the study and developed the method. Bibi Rafeena Ally‐Charles, Ede Tyrell, Rebecca Khatun, Richard Lall, Bibi Yassin, Martin King, Devi Rajnarine, Basil Dey and Charlan Abrams prepared the extracts, performed the phytochemical screening and antibacterial susceptibility testing, curated the data and wrote the initial draft of the manuscript. Charlan Abrams, Rebecca Khatun, Richard Lall, Bibi Yassin, Martin King and Devi Rajnarine prepared all the media and conducted the sterility testing. Narita Singh and Andrew Hutson conducted the formal data analysis, and Bibi Rafeena Ally‐Charles and Karishma Jeeboo performed the MIC and prepared the final manuscript.

## Funding

No funding was received for this manuscript.

## Data Availability

The data that support the findings of this study are available from the corresponding author upon reasonable request.
